# Hydrofluoric Acid Cutaneous Burns: A Systematic Review of Emergency Management and General Surgical Sequelae

**DOI:** 10.7759/cureus.104750

**Published:** 2026-03-06

**Authors:** Andrew Kelly, Sophia Chan, Matthew J Wood

**Affiliations:** 1 General Surgery, Cairns Hospital, Cairns, AUS; 2 General Surgery, Sunshine Coast University Hospital, Birtinya, AUS

**Keywords:** burn injury, chemical burns, hydrofluoric acid, skin graft, surgical treatment after burn

## Abstract

Hydrofluoric acid (HF) is a highly corrosive and toxic chemical capable of causing deep tissue injury and life-threatening systemic electrolyte disturbances. This study systematically reviews reported cases of dermal HF burns over the past decade, with emphasis on immediate management, systemic toxicity, surgical intervention, and clinical outcomes. A systematic review was conducted in accordance with Preferred Reporting Items for Systematic Reviews and Meta-Analyses (PRISMA) guidelines. Thirteen studies comprising individual case reports and one retrospective case series (N=29 patients) were included. HF concentrations ranged from dilute household preparations to highly concentrated industrial exposures, with total body surface area (TBSA) involvement ranging from <5% to 91%. Mild exposures were successfully managed with topical calcium gluconate gel or soaking techniques without significant systemic complications. Severe cases were associated with profound hypocalcemia, hypomagnesemia, hyperkalemia, ventricular dysrhythmias, metabolic acidosis, and shock. Surgical intervention, including debridement and skin grafting, was necessary primarily in patients with extensive or delayed-recognition injuries. Mortality was reported in two cases involving significant TBSA and systemic toxicity. Continued systematic reporting is necessary to refine treatment strategies and improve clinical outcomes.

## Introduction and background

Hydrofluoric acid (HF) is a highly corrosive and toxic inorganic acid used globally in industrial processes, including semiconductor etching, glass manufacturing, electroplating, and metal cleaning. Although classified chemically as a weak acid, HF causes disproportionately severe injury due to its unique ability to penetrate tissues and dissociate into fluoride ions, which bind calcium and magnesium. This mechanism results in progressive liquefactive necrosis and systemic electrolyte derangements that distinguish HF burns from other chemical injuries [1-3].

Unlike strong acids that cause superficial coagulation necrosis, HF rapidly traverses the dermal barrier, allowing fluoride ions to diffuse into deeper tissues and the systemic circulation. Clinically, patients may present with pain that is out of proportion to visible skin findings, and in some cases, erythema or tissue damage may be delayed for several hours following exposure [1,2]. Systemic fluoride absorption results in binding to calcium and magnesium, forming insoluble complexes that cause functional hypocalcemia and hypomagnesemia with associated tissue injury. The resulting calcium depletion impairs sodium-potassium adenosine triphosphatase pump activity, leading to hyperkalemia and significant electrolyte disturbances that predispose to cardiac arrhythmias, including QTc prolongation, polymorphic ventricular tachycardia, T-wave elevation, and QRS widening [1,4]. The discordance between early cutaneous appearance and evolving systemic toxicity presents a significant diagnostic and therapeutic challenge.

Early literature describing HF burns emphasized both the unpredictability of tissue injury and the importance of rapid intervention. Kirkpatrick and colleagues provided one of the foundational reviews of HF dermal injuries, outlining the pathophysiology and spectrum of clinical presentation [2]. They subsequently proposed an algorithmic management strategy centered on immediate irrigation, early calcium supplementation, and close biochemical monitoring [3]. These recommendations continue to inform contemporary practice; however, much of the supporting evidence originates from small case series and isolated reports.

Beyond dermal injury, HF exposure through ingestion or inhalation has also demonstrated the acid’s profound systemic toxicity. Kao et al. described cases of concentrated HF ingestion resulting in delayed but severe metabolic disturbances and fatal outcomes, underscoring the insidious nature of fluoride-mediated poisoning [4]. Collectively, these reports highlight a central theme in HF injury: the severity of toxicity often cannot be predicted solely by initial clinical findings.

Despite decades of clinical recognition, there remains limited evidence for accurate prognostication and the extent of surgical intervention required following HF dermal burns. Given the continued industrial use of HF and the potentially catastrophic consequences of exposure, a contemporary synthesis of reported cases is warranted.

The aim of this study was to systematically review reported cases of HF dermal burns over the past decade, with a focus on immediate management strategies, systemic toxicity, surgical intervention requirements, and patient outcomes.

## Review

Methods

A systematic review was conducted to evaluate reported cases of dermal HF burns over the preceding 10-year period, with particular focus on immediate management strategies, systemic toxicity, surgical intervention, and clinical outcomes. The review was performed in accordance with Preferred Reporting Items for Systematic Reviews and Meta-Analyses (PRISMA) guidelines.

A structured literature search was performed in PubMed (MEDLINE), Embase, and the Cochrane Library. The search was conducted on February 15, 2026, and included studies published between January 1, 2016, and February 15, 2026. Controlled vocabulary terms and free-text keywords were combined to maximize sensitivity. In PubMed, medical subject headings or MeSH and title/abstract search fields were used. The exact PubMed search strategy is presented in the Appendix. Equivalent controlled vocabulary and keyword adaptations were used in Embase. No filters were applied beyond language (English) and publication date.

**Figure 1:**
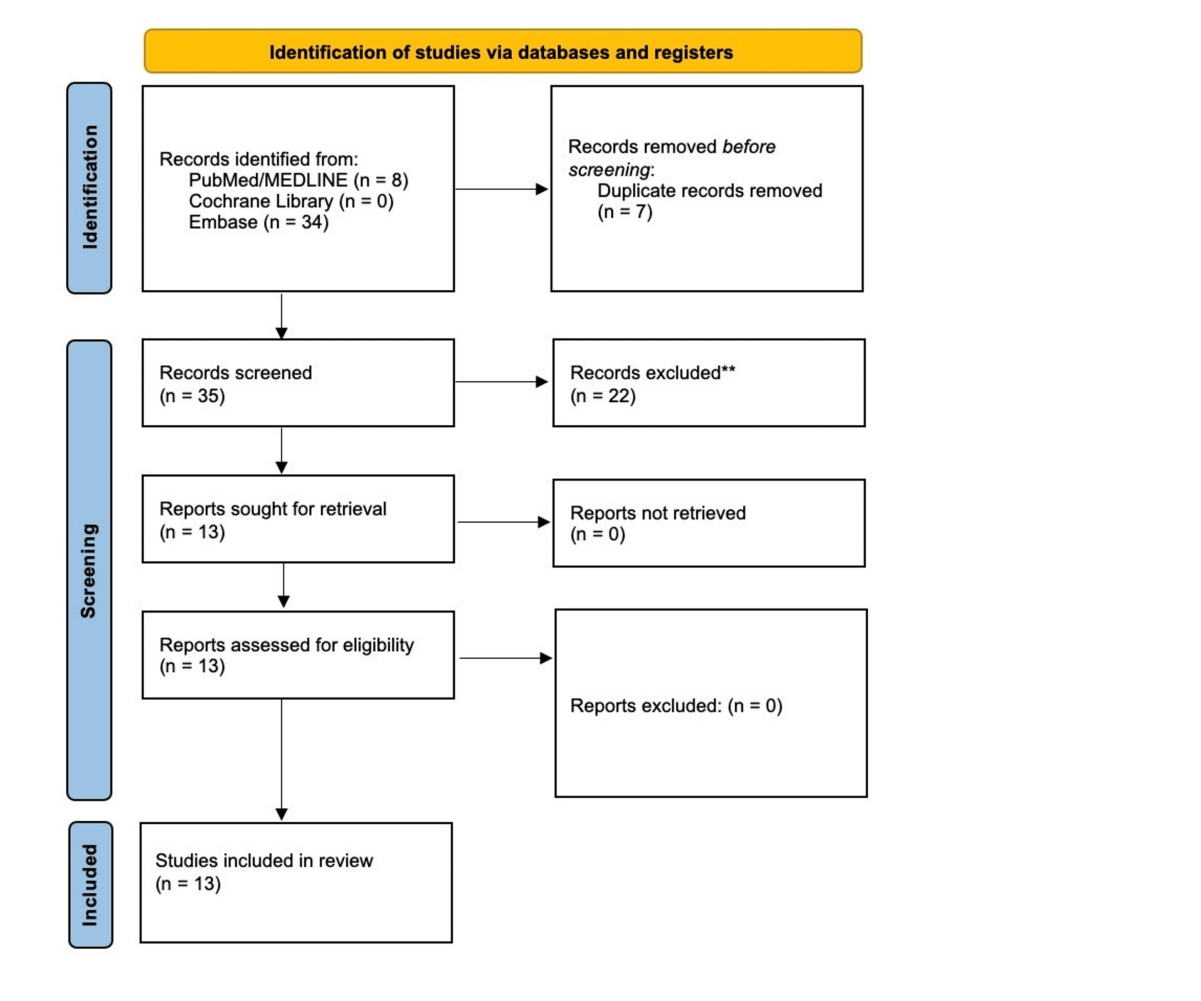
PRISMA flow diagram of literature search and study selection A total of 42 records were identified (PubMed: 8, Embase: 34, and Cochrane: 0). After removing 7 duplicates, 35 records were screened. **Studies were excluded based on predefined criteria, including non-dermal exposure, non-human data, lack of primary case information, publication outside January 1, 2016, and February 15, 2016, and insufficient management or outcome data. Thirteen studies were included in the final analysis. PRISMA, Preferred Reporting Items for Systematic Reviews and Meta-Analyses.

The search yielded 8 records from PubMed, 34 from Embase, and 0 from the Cochrane Library, resulting in a total of 42 records. Seven duplicate records were identified and removed, leaving 35 unique studies for screening. Titles and abstracts were reviewed for relevance, followed by a full-text review of potentially eligible studies. Thirteen studies met the predefined inclusion criteria and were included in the final qualitative synthesis. The study selection process is summarized in Figure 1.

Studies were eligible for inclusion if they involved human subjects with dermal or cutaneous exposure to HF, were designed as case reports/case series/randomized control trials/cohort studies, were published within the defined 10-year study period, and provided sufficient detail regarding clinical management and outcomes. Studies were excluded if they involved isolated inhalational or ingestion exposures without dermal involvement, non-human data, narrative reviews without original patient-level data, publication outside the study timeframe, or insufficient clinical detail. 

Data were extracted manually from full-text articles using a predefined standardized framework. Extracted variables included study characteristics; patient demographics; exposure details such as HF concentration, total body surface area (TBSA) affected, anatomical location, and mechanism of exposure; immediate management, including irrigation and calcium supplementation modality and dosing; monitoring strategies, including electrolyte measurement and electrocardiography; evidence of systemic toxicity, including hypocalcemia, hypomagnesemia, hyperkalemia, metabolic acidosis, arrhythmias, intensive care admission, renal replacement therapy, or extracorporeal membrane oxygenation (ECMO); surgical interventions such as debridement, excision, grafting, or amputation; and final clinical outcomes, including survival, complications, length of hospital stay, and functional recovery. When case series were included, patient-level data were extracted where available.

Given the heterogeneity of case reports and the absence of comparative cohorts, quantitative meta-analysis was not performed. Instead, findings were synthesized descriptively, with emphasis on patterns of immediate management, frequency and severity of systemic toxicity, surgical intervention requirements, and patient outcomes.

Results

The literature search identified 42 records (PubMed: 8, Embase: 34, and Cochrane: 0). After removal of 7 duplicates, 35 studies underwent title and abstract screening. Following full-text review, 13 studies met the predefined inclusion criteria and were included in the final qualitative synthesis (Table 1). These consisted of 12 case reports and 1 retrospective case series comprising 29 patients [5-17].

**Table 1:** Characteristics, surgical management, and outcomes of patients with dermal hydrofluoric acid burns included in the systematic review (2016-2026) ARDS, acute respiratory distress syndrome; Ca, calcium; CaCl, calcium chloride; CRRT, continuous renal replacement therapy; ECMO, extracorporeal membrane oxygenation; HF, hydrofluoric acid; ICU, intensive care unit; iCa, ionized calcium; IV, intravenous; NPWT, negative pressure wound therapy; NR, not reported; TBSA, total body surface area; VF, ventricular fibrillation.

Study	Design (N)	Exposure characteristics	Immediate management	Systemic toxicity/unusual consequences	Surgical management	Outcome
Pan et al. (2025) [5]	Retrospective case series (N=29)	HF: 0.03%-49% and mean TBSA: 1.60%±2.93%	Calcium gluconate soaking, nail removal (N=1, 3.4%), and debridement (N=4, 13.8%)	1 ICU admission, no significant electrolyte imbalance, and no fatalities	Debridement in four (13.8%) patients	All survived, effective for mild burns
Ramesh et al. (2025) [6]	Case report (N=1)	HF unknown concentration, dermal HF burn, TBSA: 10%, and involved face	Topical calcium gluconate, intravenous calcium gluconate 20 mL 10%, and nebulized calcium gluconate	Severe systemic toxicity and rhabdomyolysis	NR	Fatal
Hu et al. (2024) [7]	Case report (N=1)	35% HF and 91% TBSA	ICU care, IV + percutaneous calcium, blood purification, and tracheotomy	Shock, severe hypocalcemia, metabolic acidosis, and respiratory failure	Early debridement, multiple grafting procedures, NPWT, and Meek grafting	Survived, functional recovery
Meaney et al. (2023) [8]	Case report (N=1)	10% HF, ~5% TBSA, and upper extremities	Irrigation, topical calcium gel, Bier block, and intradermal calcium	No electrolyte abnormalities and pain out of proportion	None	Healed without surgery
Prasad et al. (2021)[9]	Case report (N=1)	60% HF and 15% full-thickness burns	Topical and subcutaneous calcium; IV CaCl, Mg; insulin-glucose; and CRRT	Severe metabolic acidosis and VF cardiac arrest	NR	Fatal
Hoffmann et al. (2021) [10]	Case report (N=1)	Dermal HF burn and size NR	Topical calcium gluconate gel	No severe systemic toxicity reported	None reported	Survived
Yu et al. (2020) [11]	Case report (N=1)	30% TBSA and 45%-50% HF	Rapid electrolyte therapy and supportive care	Significant electrolyte disturbance	Debridement and tangential excision	Complete wound healing
Fang et al. (2019) [12]	Case report (N=1)	15% HF, 60% TBSA, and inhalation injury	IV calcium (large cumulative doses) and ECMO	VF ×9, iCa 0.192 mmol/L, and ARDS	Skin grafting	Survived, prolonged admission
Little et al. (2018) [13]	Case report (N=1)	3%-7% HF + sulfuric acid and hand	Prehospital calcium source (yogurt) and ED calcium gel	No major systemic toxicity	None	Pain resolved within 24 hours
Pu et al. (2017) [14]	Case report (N=1)	10% HF + nitric acid and ~60% TBSA	IV calcium bolus + infusion, Mg infusion, CRRT, and ECMO	Severe hypocalcemia and metabolic instability	Skin grafting (×2)	Survived
Zhang et al. (2017) [15]	Case report (N=1)	53% HF and face/neck/nasal cavity	Fluids, electrolyte replacement, and CRRT	Hypocalcemia and myocardial injury	NR	Survived
Steverlynck et al. (2017) [16]	Case report (N=1)	Inhalation exposure (HF + nitric acid)	Decontamination, supportive care, and calcium therapy	Delayed respiratory symptoms	N/A	Recovered
Hung and Yang (2016) [17]	Case report (N=1)	Unknown concentration and leg exposure	Initial misdiagnosis and later calcium therapy	Deep tissue necrosis and delayed recognition	Repeated debridement and grafting	Improved

HF concentrations ranged from dilute household preparations (3%-7%) to highly concentrated industrial exposures exceeding 50% [8,11,13,15]. TBSA involvement varied widely, from localized hand exposures involving less than 5% TBSA [8,13] to extensive injuries affecting 60%-91% TBSA [7,12,14]. Mechanisms of injury included occupational industrial splashes, immersion in acid-containing solutions, mixed acid exposures, and inhalational injury associated with dermal contact [12,14,16].

Mild and localized exposures were generally managed using topical calcium gluconate gel or calcium soaking techniques without major complications. In the retrospective case series of 29 patients, all were treated with calcium gluconate soaking without the need for intra-arterial or subcutaneous injections, and no significant electrolyte abnormalities or mortality were reported [5]. Similarly, smaller TBSA exposures involving the upper extremities responded to irrigation, topical calcium therapy, and, when required, regional intravenous (Bier block) or intradermal calcium injections, with resolution of pain and no surgical intervention [8,10,13]. Pain out of proportion to visible injury was frequently described and commonly used as a clinical indicator to guide escalation of calcium therapy [8,13].

In contrast, severe systemic toxicity was observed predominantly in patients with larger TBSA involvement or higher concentration exposures. Profound hypocalcemia was documented in several cases, with ionized calcium levels reported as low as 0.192 mmol/L [12,14]. Ventricular dysrhythmias, including recurrent ventricular fibrillation (VF), were described in multiple severe cases [9,12]. One patient experienced nine episodes of VF within the first two hours of presentation [12]. Metabolic acidosis, hypomagnesemia, hyperkalemia, shock, and respiratory failure were also reported in extensive injuries [7,9,12,14,15]. Continuous cardiac monitoring and serial electrolyte measurements were commonly employed in moderate-to-severe cases [8,9,12,14].

Advanced supportive therapies were required in select patients with life-threatening systemic toxicity. Continuous renal replacement therapy (CRRT) was used in cases with refractory electrolyte disturbances or suspected ongoing fluoride toxicity [9,14,15]. ECMO was implemented in patients with hemodynamic instability or acute respiratory distress syndrome secondary to inhalational injury [12,14]. Large cumulative doses of intravenous calcium gluconate were administered in severe cases, with one report documenting a total of 343 g during the course of treatment [12].

Surgical intervention was used primarily in patients with deep partial- or full-thickness burns, extensive TBSA involvement, or delayed recognition of HF as the causative agent. Early debridement and tangential excision were performed in severe high-concentration exposures [7,11]. Several patients required staged surgical management, including negative pressure wound therapy and split-thickness skin grafting [7,12,14,17]. In contrast, patients with limited surface area burns who received early calcium therapy did not require operative intervention [5,8,10,13].

Mortality was reported in severe cases involving systemic toxicity; however, TBSA was not predictive, with fatalities reported as low as 10% TBSA in the case reports included. However, previous reports include as low as 2.5% [6]. One patient with 15% full-thickness burns developed recurrent cardiac arrest despite aggressive electrolyte correction and renal replacement therapy and did not survive [9]. However, survival with meaningful functional recovery was achieved in several extensive injuries when prompt multidisciplinary care was instituted [7,12,14].

Overall, the findings demonstrate substantial heterogeneity in exposure severity and clinical course. While mild dermal exposures have been effectively managed with topical calcium therapy alone, severe cases carry a high risk of systemic toxicity and may require advanced critical care support and surgical intervention.

Overall methodological quality was moderate to high, with the majority of studies demonstrating low risk of bias; limitations were primarily related to incomplete follow-up and variability in reporting of intervention details (Table 2).

**Table 2:** Methodological quality assessment of included studies using adapted Joanna Briggs Institute critical appraisal criteria Overall, the appraisal was categorized as low or moderate risk of bias based on completeness of reporting and follow-up. Yes, criterion fulfilled; Partial, incompletely reported; No, not reported.

Study	Clear patient description	Exposure measurement valid	Intervention clearly described	Post-intervention outcomes reported	Adverse events reported	Follow-up adequate	Overall appraisal
Pan et al. (2025) [5]	Yes	Yes	Yes	Yes	Yes	Yes	Low
Ramesh et al. (2025) [6]	Yes	Yes	Partial	Yes	Yes	No	Moderate
Hu et al. (2024) [7]	Yes	Yes	Yes	Yes	Yes	Yes	Low
Meaney et al. (2023) [8]	Yes	Yes	Yes	Yes	Yes	Yes	Low
Prasad et al. (2021) [9]	Yes	Yes	Yes	Yes	Yes	No	Moderate
Hoffmann et al. (2021) [10]	Yes	Yes	Yes	Yes	Yes	Partial	Low
Yu et al. (2020) [11]	Yes	Yes	Yes	Yes	Yes	Yes	Low
Fang et al. (2019) [12]	Yes	Yes	Yes	Yes	Yes	Yes	Low
Little et al. (2018) [13]	Yes	Yes	Yes	Yes	Yes	Yes	Low
Pu et al. (2017) [14]	Yes	Yes	Yes	Yes	Yes	Yes	Low
Zhang et al. (2017) [15]	Yes	Yes	Yes	Yes	Yes	Yes	Low
Steverlynck et al. (2017) [16]	Yes	Yes	Partial	Yes	Yes	Yes	Moderate
Hung and Yang (2016) [17]	Yes	Partial	Yes	Yes	Yes	Yes	Moderate

Discussion

This systematic review synthesizes contemporary case reports and case series describing dermal HF burns over the past decade, with emphasis on immediate management strategies, systemic toxicity, surgical intervention, and clinical outcomes. The findings reinforce the distinctive and potentially catastrophic nature of HF injury, characterized by a disconnect between initial cutaneous appearance and evolving metabolic and systemic derangement [1-3].

A consistent theme across the included studies is the rapid tissue penetration of fluoride ions and the resulting electrolyte disturbances, particularly hypocalcemia, hypomagnesemia, and hyperkalemia. Severe systemic toxicity was observed primarily in patients with larger TBSA involvement or higher acid concentrations [7,11,12,14,15]. Several cases demonstrated profound hypocalcemia with ventricular dysrhythmias and cardiac arrest, sometimes occurring within hours of exposure [9,12,14]. In one extreme case involving 60% TBSA exposure, VF occurred nine times within the first two hours, with an ionized calcium level of 0.192 mmol/L [12]. Similar critical hypocalcemia was reported in another 60% TBSA exposure requiring aggressive calcium replacement and extracorporeal support [14]. These findings reflect the fluoride-mediated chelation of calcium and magnesium described in foundational toxicology literature [1,4].

Importantly, systemic toxicity was not exclusively limited to extensive burns. Cases involving smaller surface areas still demonstrated significant metabolic complications, particularly when treatment was delayed or recognition was incomplete [6,17]. The phenomenon of pain out of proportion to visible injury was repeatedly described and remains a critical clinical indicator of ongoing fluoride ion activity within deeper tissues [8,13,17]. In several reports, visible cutaneous findings evolved over days to weeks despite early presentation, underscoring the potential for progressive tissue injury [8,17].

Calcium supplementation remains the cornerstone of management, consistent with historical algorithmic recommendations [3]. Across the included studies, a range of administration strategies was employed depending on severity. Topical calcium gluconate gel or soaking techniques were effective in mild cases [5,10,13]. In a retrospective case series of 29 patients, calcium gluconate soaking was successfully used in all cases without significant electrolyte disturbances or mortality [5]. For more severe localized exposures, escalation to intradermal injections and regional intravenous (Bier block) techniques provided rapid pain relief and prevented progression [8]. Pain resolution was frequently used as a surrogate marker for adequate fluoride neutralization [8,13].

In cases of severe systemic toxicity, large cumulative doses of intravenous calcium were required [7,12,14]. One patient received 343 g of calcium gluconate during the course of treatment [12]. Extracorporeal therapies were employed in the most critical presentations. CRRT was used to manage refractory electrolyte disturbances and remove circulating fluoride ions [9,14,15], while ECMO was implemented in patients with cardiac instability or respiratory failure [12,14]. These advanced interventions underscore the potential severity of HF exposure and the need for multidisciplinary critical care management in extensive or complicated cases.

Surgical intervention was required in patients with extensive deep partial- or full-thickness burns or in cases of delayed recognition. Early debridement and excision were performed in large TBSA injuries to prevent wound progression [7,11]. Staged procedures, including negative pressure wound therapy and skin grafting, were reported in multiple severe cases [7,12,14,17]. In contrast, patients with smaller exposures who received timely calcium therapy often avoided operative management [5,8,10,13]. These findings suggest that early recognition and aggressive neutralization may mitigate progression to deep tissue necrosis and reduce the need for surgical intervention.

Unusual or emerging complications were also identified. Rhabdomyolysis was reported in association with systemic HF toxicity, representing a potentially underrecognized manifestation [6]. Myocardial injury was described in a patient with high-concentration facial exposure [15]. Inhalational injury combined with dermal burns resulted in acute respiratory distress syndrome requiring ECMO support [12]. These reports highlight that the systemic consequences of HF exposure may extend beyond classical electrolyte disturbances.

Despite aggressive intervention, mortality remains a risk in severe HF burns, particularly in cases involving significant TBSA or delayed treatment. A fatal case involving 15% full-thickness burns demonstrated recurrent VF resulting in cardiac arrest despite intensive correction of electrolyte abnormalities, renal replacement therapy, and defibrillation [9]. However, other reports demonstrate that survival with meaningful functional recovery is achievable even after extensive injury when prompt multidisciplinary management is instituted [7,12,14].

This review has several limitations. The available evidence consists exclusively of case reports and case series, introducing publication bias and limiting generalizability. Variability in reporting of electrolyte values, calcium dosing regimens, timing of intervention, and follow-up outcomes restricts quantitative comparison. Additionally, heterogeneity in exposure concentration, mechanism, and prehospital management complicates direct comparisons between cases. Nonetheless, given the rarity and severity of HF burns, case-based evidence remains an important source of clinical insight.

HF dermal burns represent a uniquely dangerous chemical injury characterized by deep tissue penetration and potentially life-threatening systemic toxicity. Early recognition, prompt calcium administration, vigilant metabolic monitoring, and timely surgical intervention when necessary are critical determinants of outcome. Severe cases may require advanced critical care support, including renal replacement therapy and ECMO. Continued systematic reporting and synthesis of contemporary cases are essential to refine management strategies and improve patient outcomes, even in the development of a risk calculator to predict ICU stay and mortality.

## Conclusions

HF dermal burns represent a uniquely hazardous form of chemical injury characterized by rapid tissue penetration and the potential for severe, life-threatening systemic toxicity. Unlike other corrosive agents, the severity of HF exposure is not reliably predicted by the initial appearance of the skin, and even seemingly minor burns may progress to significant electrolyte derangements and cardiac instability. This systematic review of contemporary case reports and case series highlights the central importance of early recognition, prompt decontamination, and aggressive calcium supplementation as the foundation of management. Pain out of proportion to visible injury remains a critical clinical warning sign. While mild exposures may respond to topical or soaking calcium preparations, more severe cases frequently require escalation to intradermal or intravenous calcium administration, intensive electrolyte monitoring, and multidisciplinary critical care involvement. Extensive injuries may necessitate surgical intervention, including debridement and skin grafting, and in rare but severe presentations, advanced supportive therapies such as continuous renal replacement therapy and extracorporeal membrane oxygenation may be required.

Although outcomes in severe cases have improved with modern critical care and burn management, mortality remains a risk, particularly in patients with large TBSA involvement or delayed treatment. Given the rarity of HF burns and the predominance of case-based evidence, continued systematic reporting is essential to refine management algorithms and improve patient outcomes.

## Appendices

**Table 3:** Search terms and search strategy, demonstrating terms used for Pubmed, Embase, and Cochrane Library

Database	Search strategy
PubMed (MEDLINE)	("Hydrofluoric Acid"[Mesh] OR hydrofluoric acid[tiab] OR hydrogen fluoride[tiab] OR HF acid[tiab] OR fluoride poisoning[tiab] ) AND ("Burns, Chemical"[Mesh] OR burn*[tiab] OR chemical burn*[tiab] OR dermal exposure[tiab] OR cutaneous exposure[tiab] OR skin[tiab] ) AND ("Case Reports"[Publication Type] OR case report*[tiab] OR case series[tiab])
Embase	('hydrofluoric acid'/exp OR 'hydrofluoric acid':ti,ab OR 'hydrogen fluoride':ti,ab) AND ('chemical burn'/exp OR burn*:ti,ab OR dermal:ti,ab OR cutaneous:ti,ab) AND ('case report'/exp OR 'case series':ti,ab)
Cochrane Library	("hydrofluoric acid" OR "hydrogen fluoride" OR "fluoride poisoning") AND (burn* OR "chemical burn*" OR dermal OR cutaneous OR skin)
